# Pyramidal metamaterial absorber for mode damping in microwave resonant structures

**DOI:** 10.1038/s41598-020-76433-3

**Published:** 2020-11-09

**Authors:** Nassim Chikhi, Andrea Passarelli, Antonello Andreone, Maria Rosaria Masullo

**Affiliations:** 1 Physics Department, University of Naples “Federico II”, Naples, 80126 Italy; 2 INFN Naples Unit, Naples, 80126 Italy

**Keywords:** Electrical and electronic engineering, Electronics, photonics and device physics

## Abstract

In many resonant structures the damping of parasitic or higher order modes is indispensable to guarantee a correct and stable performance. This is particularly true in the microwave region in case of cavities or other resonant systems operating in accelerating structures, where the mitigation of spurious resonance effects is mandatory to achieve high quality particle beams. We present the results on the mode suppression in a real pillbox cavity by inserting a properly designed pyramidal metamaterial that acts as light, small volume damper for specific resonances in the range 3–4 GHz, only slightly perturbing other intrinsic modes. Measurements of the cavity response without and with the metamaterial absorber are presented and compared with full wave simulations. Field distribution for the pillbox intrinsic modes under scrutiny is also presented, showing that damping induced by the metamaterial critically depends on its relative position inside the cavity.

## Introduction

Since the realisation of the first negative refraction medium using a metamaterial (MM)^1,2^, motivated by Veselago’s prediction of new type of materials that can exhibit exotic features nonexistent in nature^3^, considerable efforts have been spent worldwide within the scientific community to fruitfully use these engineered structures in real world applications such as antennas, absorbers, superlenses, wave cloaks. MMs can be electromagnetically treated as effective media with tailored permittivity and permeability, since their macroscopic response depends on sub-wavelength units cell acting as artificial “atoms” with properties depending on design rather than material constituents.

During the last years, MMs made of layered metal-dielectric elements received a great deal of attention for the realization of highly absorptive structures^4^. The generally accepted idea underneath the mechanism of absorption is that by tuning the effective dielectric permittivity and magnetic permeability independently, it is possible to realise impedance matching to free space^5^ and minimise the reflection. At specific conditions in fact a collective electromagnetic resonance can be realised and total absorption achieved, mainly imposed by the geometry of the structure. During the years, many metamaterial planar designs have been proposed, usually consisting of lattice of conducting unit cells and a ground plane intercalated with an absorptive layer. Square and circular patches^6,7^, cut and coiled wires^8,9^, closed and split rings^10,11^, cross-shaped resonators^12^ and many other geometries have been presented, differing mostly on the absorption level and frequency band only. Since in all these structures the process of absorption is based on the presence of intrinsic (geometrical) resonances, their operational bandwidth is usually narrow, not exceeding some percent with respect to the central frequency in most of the cases. There are however many applications where broadband absorption is required, like in energy harvesting for civil applications, military stealth or in challenging devices like those mounted on beam pipes of high luminosity accelerator facilities. The expansion of the absorption bandwidth can be realised by exploiting structures with multiple resonances^13^ or properly blending different resonators put together on the same plane^14^ or distributed on different layers^15^; however, the structure design is often extremely complex and there are usually size constrains effectively limiting the number of resonators that can be practically located. Moreover, the use of too many typologies of resonant unit cells intrinsically represents a conflicting approach in trying to expand as much as possible the operational bandwidth and keep at the same time a high level of absorption^16^.

To overcome the narrow absorption band various metastructures have been investigated. Broadband MM absorbers based on multilayered cells have been proposed and experimentally demonstrated with an absorptivity level larger than $90\%$ in the microwave region (in the 8–14 GHz range)^17^, limited by the overall height of the structure only. The basic concept here grounds in the design of meta-structures consisting of sub-wavelength multiple resonators (“sub-cells”) having slightly different geometrical parameters and therefore adjacent intrinsic resonances, so that when stacked along the vertical direction they present a continuous and wide frequency response without decreasing the absorption strength in the spectrum. In the last years, arrays having multi-sized pyramidal unit cells have been proposed to widen the frequency bandwidth (2.3–40 GHz) by spectral overlapping; an absorption in the order of $80\%$ has been obtained^18^. Starting from the same MM absorbers, the full use of both the fundamental and ${\hbox {TM}}_{210}$ harmonics excited by diffraction of waves at each layer edges allowed a band from 7 to 18 GHz and an absorption larger than $90\%$^19^. Alternatively, a pyramidal periodic structure comprising carbon nanotubes, spherical carbonyl iron, and silicone rubber gave a bandwidth from 2 to 40 GHz and an absorption larger than $90\%$^20^.

Many different technologies are currently in use for manufacturing wideband absorbers. Frequency Selective Surfaces posed between two Carbonyl Iron filled Silicon Rubber(CISR) sheets have been proved to work in the range from 4 to 8.2 GHz with $-10$ dB attenuation^21^. Polypyrrole/NiZn ferrite nanocomposites were successfully prepared for working between 7 and 13 GHz and an attenuation up to $-30$ dB^22^. Reduced graphene oxide/${\hbox {La}}_{0.7}{\hbox {Sr}}_{0.3}{\hbox {MnO}}_{3}$ (rGO/LSMO) composites exhibited high values of reflection loss over a wide frequency range from 11.9 to 16.9 GHz, with a peak of $-47.9$ dB^23^. Wideband folded-dipole antenna array backed through a quarter-wavelength spacer by a metal ground allowed for a 2.7–8.2 GHz bandwidth and a maximum attenuation of $-15$ dB^24^.

Using resonator vertical arrangement, light-harvesting structures have been developed based on periodic taper arrays constructed by an alumina–chrome multi-layered MM on a gold substrate, with unprecedented broadband absorption (larger than 90%) over almost the entire solar spectrum^25^. Similarly, in the infrared region, thin multilayered metamaterials consisting of periodic arrays of sawtoothed anisotropic slabs have been used for designing photovoltaic devices and thermal emitters^26^ with absorptivity higher than $95\%$ in the range from 50 to 100 THz.

However, tailored applications require an optimization between the bandwidth, the absorbance level, the space availability and also specific characteristics on used material due to environment requirements. Many of the previously described structures occupy relatively big volumes or have large effective thickness or present complexities in the fabrication to achieve a broadband absorption. In this work, we propose a properly designed single multi-layered tapered electromagnetic (EM) absorbing structure to be used as damping element for undesired intrinsic modes in resonant structures. By exploiting its particular properties, we realise an efficient damper in the microwave region having a small overall “electrical volume” (volume normalised to the cubic wavelength) without sacrificing the level of absorption (larger than 90%). This type of MM can be an excellent alternative to existent conventional techniques used as absorbers of specified modes inside resonant structures, namely RF-fingers and natural materials such as ferrites^27^. The first technology becomes cumbersome and cannot be effectively employed in a very high frequency regime (above 30 GHz), the second one faces a serious problem in a high temperature environment such as inside collimators used in accelerating structures, since the large degassing rate induced during the machine operation might degrade the beam vacuum quality. Other higher order modes (HOMs) damping strategies have been also proposed in the past, based on the concept of “photonic” cavities. A monomodal open resonator can be easily created by introducing a lattice defect in a periodically^28^ or aperiodically ordered dielectric^29^ or hybrid (metallo-dielectric)^30^ arrangement. Point-defected cavities are based on the remarkable properties to isotropically reflect waves in certain ranges of frequencies called “band gaps”^31^, while allowing other frequencies to propagate. Major drawbacks in these structures are size, thermal load, and mode suppression critically dependent on the design.

In the following, we present the design and realisation of a metamaterial based on a multilayered truncated pyramid (or square frustum) and its performance as mode damper when inserted in a microwave pillbox, taken as “model” resonant cavity. We show experimentally, and with the support of full wave simulations, that the proposed structure fully meets all the general requirements of absorbing devices: strong absorption of EM waves, broad bandwidth response, low weight and small volume compared to the cubic wavelength. A pyramidal absorber can be used to remove specific unwanted modes, only slightly affecting other intrinsic resonances, with mode suppression efficiently depending on its relative position inside the cavity. This approach may represent a novel and efficient way to damp different typologies of modes in microwave cavities. Results presented here can be readily applied to other classes of resonant structures, and easily extended also at higher frequencies, up to the mm and sub-mm wave regions.

## Results

**Figure 1:**
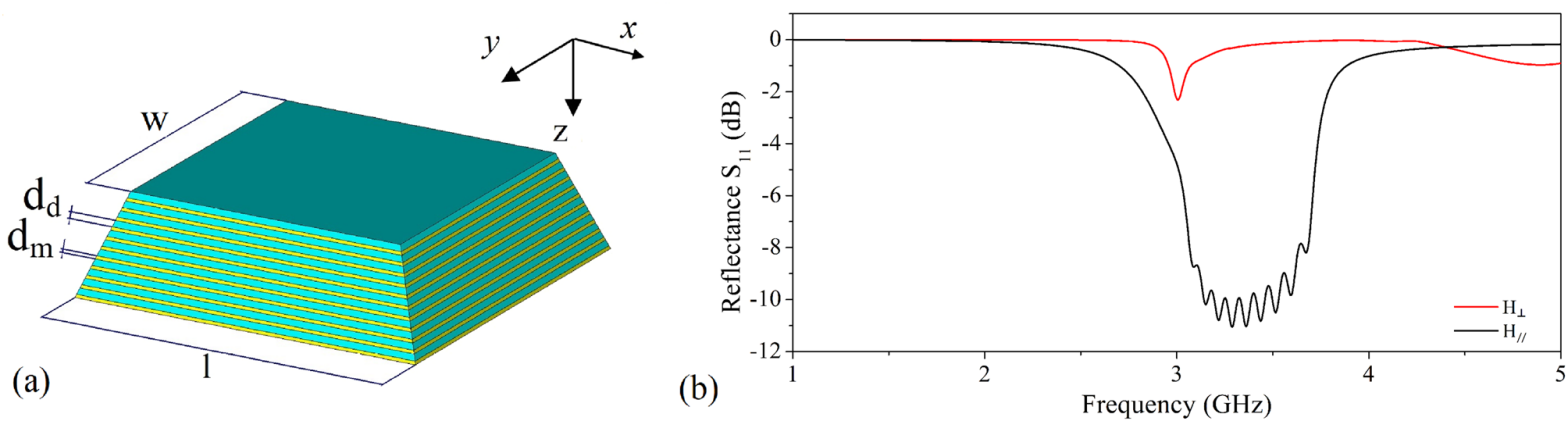
(**a**) Pictorial representation of the pyramidal structure and (**b**) simulated ${\text {S}}_{11}$ scattering parameter obtained with magnetic field **H** parallel (black curve) and perpendicular (red curve) to the pyramid base respectively. In our configuration $w=19{\hbox { mm}}$, $l=24{\hbox { mm}}$, $d_m=0.2{\hbox { mm}}$ and $d_d = 0.4{\hbox { mm}}$.

We designed a pile of stacked metal (Copper)-dielectric (FR-4) layers having thickness $d_m$ and $d_s$ respectively, each one with its own resonant frequency and geometry, to form a whole structure having the shape of a truncated pyramid (Fig. 1a). A series of these individual resonators operating at different adjacent frequencies can realise a broadband absorber^5^.

Assuming a thickness for each layer much smaller than the wavelength, the multilayered structure behaves as an homogeneous medium having an effective complex permittivity and permeability and an overall EM response determined by the geometry of the sub-cells. Of course, the presence of losses in the conducting and dielectric layers is a mandatory requirement for the design of an efficient absorber.

In order to study and test the efficiency of a pyramidal absorber as wave damper for microwave resonant structures, we used a cylindrical pillbox cavity working in the range 1–4 GHz. In the empty cavity, the first five transverse magnetic (TM) modes are the ${\text {TM}}_{010}$, ${\text {TM}}_{011}$, ${\text {TM}}_{012}$, ${\text {TM}}_{020}$, ${\text {TM}}_{021}$, which resonate at 1.54 GHz, 2.16 GHz, 3.38 GHz, 3.56 GHz and 3.89 GHz, respectively^32^. A truncated pyramid with lower base of $24\times 24{{\hbox { mm}}^2}$, upper base of $19\times 19{{\hbox { mm}}^2}$, and height $6{\hbox { mm}}$ has been selected to show an absorption band as broad as possible in the frequency range including the last three TM cavity modes, between 3 and 4 GHz.

At first, the intrinsic EM behaviour of a single square frustum has been studied by means of numerical simulations evaluating its response to a plane wave incident from the top level along the z-direction. Electric and magnetic boundary conditions have been applied to the x–z and y–z planes. Since the pyramid bottom surface is made of copper, the wave incident on the top layer along the z-direction is blocked and thus absorption A can be simply expressed as $A\left( \omega \right) = 1-R\left( \omega \right)$, where $R\left( \omega \right) =\left| S_{11}\left( \omega \right) \right| ^2$ is the reflection coefficient and ${\text {S}}_{11}$ is the scattering parameter. The relative absorption bandwidth is defined as $W_{RAB}=2(f_{2}-f_{1})/(f_{2}+f_{1})$ with $f_{1}$ and $f_{2}$ representing the lower and upper values respectively in the frequency band where absorption exceeds 90%.

In Fig. 1b, ${\text {S}}_{11}$ is plotted for different wave incident directions with respect to the pyramid, so that the magnetic field is perpendicular, $H_{\perp }$, or parallel, $H_{\parallel }$, to the basal plane.

The red curve shows the reflection coefficient for the first case. No resonant behaviour can be observed in the frequency range of interest, the small dip present at around 3 GHz being a simulation artefact (${\text {S}}_{21}$ and ${\text {S}}_{11}$ have the same shape). This is consistent with previous studies^16^ showing that absorption diminishes with increasing the magnetic field normal component, since the EM wave can no longer effectively induce antiparallel surface currents in the MM conductive layers resulting in a drop in the magnetic flux.

The reflection coefficient when the magnetic field is parallel to the pyramid plane is represented by the black curve. In this configuration instead a broadband absorption approximately between 3 and 3.8 GHz is observed, with estimated values for the maximum absorption *A* and $W_{RAB}$ of $92\%$ and $20\%$, respectively. Simulations therefore show that, in a defined frequency interval, the larger the magnetic field component parallel to the pyramid surface, the better absorption efficiency inside the MM. Under this condition, the signal displays also a sequence of in-band ripples almost equal in number to the metal-dielectric layers forming the truncated pyramid. These oscillations prove that the bandwidth is due to the overlapping response of coupled resonators operating at adjacent frequencies, with the lower frequency determined by the base layer and the higher frequency by the top layer.

Moreover, the bandwidth and the absorption level can be tuned by adjusting the pyramid ramp (defined as the line slope between the lower and upper bases)^33^. Indeed, a steep (gentle) ramp translates in a small (large) difference between the upper and lower base dimensions which implies a narrower (wider) bandwidth. Moreover, the closer are the resonance frequencies the stronger is the coupling between adjacent resonators, increasing in such a way the overall energy storage of the resonators, and in turn the absorption ability of the MM. Therefore, the narrower is the bandwidth the higher is the pyramid signal absorption.

The absorbing bandwidth of the designed structure nearly overlaps the last three resonant modes of the cavity, namely ${\text {TM}}_{012}$, ${\text {TM}}_{020}$, ${\text {TM}}_{021}$. Outside this band, the pyramid actually behaves as a dissipative load, leaving unperturbed the ${\text {TM}}_{010}$ and ${\text {TM}}_{011}$ modes but lowering the resonance Q factor values, with 40% maximum reduction. Therefore, in the following analysis we will focus on the last three modes only. The efficiency of the pyramidal MM as mode damper is assessed measuring the cavity scattering parameters to evaluate the absorption and looking at the cavity TM mode field distributions using full wave simulations, to check if the targeted modes have been damped without creating new spurious resonances.

**Figure 2:**
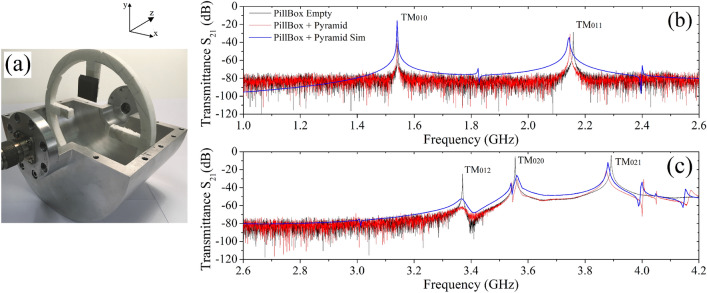
(**a**) Open view picture of the real pillbox cavity, with details of the coupling antennas and the position where the MM is placed on the dielectric support. (**b**) ${\text {S}}_{21}$ transmission parameter measurements (black curve for the empty, red curve for the loaded cavity) and simulations (blue curve) for the ${\text {TM}}_{010}$ and ${\text {TM}}_{011}$ modes. (**c**) As in (**b**), for the ${\text {TM}}_{012}$, ${\text {TM}}_{020}$, and ${\text {TM}}_{021}$ modes.

For our studies, we modelled the whole system cavity-pyramid, including a dielectric holder made of Rohacell®, inserted as shown in the picture of Fig. 2a. The support allows to place the pyramid in different positions inside the pillbox, with the metal-dielectric layers always parallel to the cavity magnetic field and with a minimal or insignificant effect on the intrinsic TM modes. We first measured the transmittance loading the cavity with one square frustum placed as visible in the open view picture (position P1, see below). The comparison between the empty and the loaded cavity cases is shown in Fig. 2b,c, black and red curves respectively. As expected, the MM clearly affects to some extent the resonances which fall within its absorbing bandwidth (Fig. 2c), leaving almost unchanged the other modes (Fig. 2b). The spectral shift of the unperturbed modes with respect to the empty cavity is mostly due to the MM effective permittivity larger than air. The magnitude of this frequency displacement is not constant, since it depends on the local EM field density in the region where the square frustum is placed inside the cavity (see discussion below). The simulated transmittance for the loaded cavity case is reported in the same figures (blue curve), showing a nice agreement between measured and numerical data.

Absorption strongly depends on the polarization of external radiation that induces a trapped electromagnetic wave in between the metallic layers. Moreover, the stronger is the EM local field intensity, the larger is the overall effect on the damping. For this reason, from the knowledge of the field distribution at each cavity resonant frequency included in the MM operational band, one can estimate the best position of a single pyramid optimizing the coupling with the cavity and therefore its potential mode disruption.

**Figure 3:**
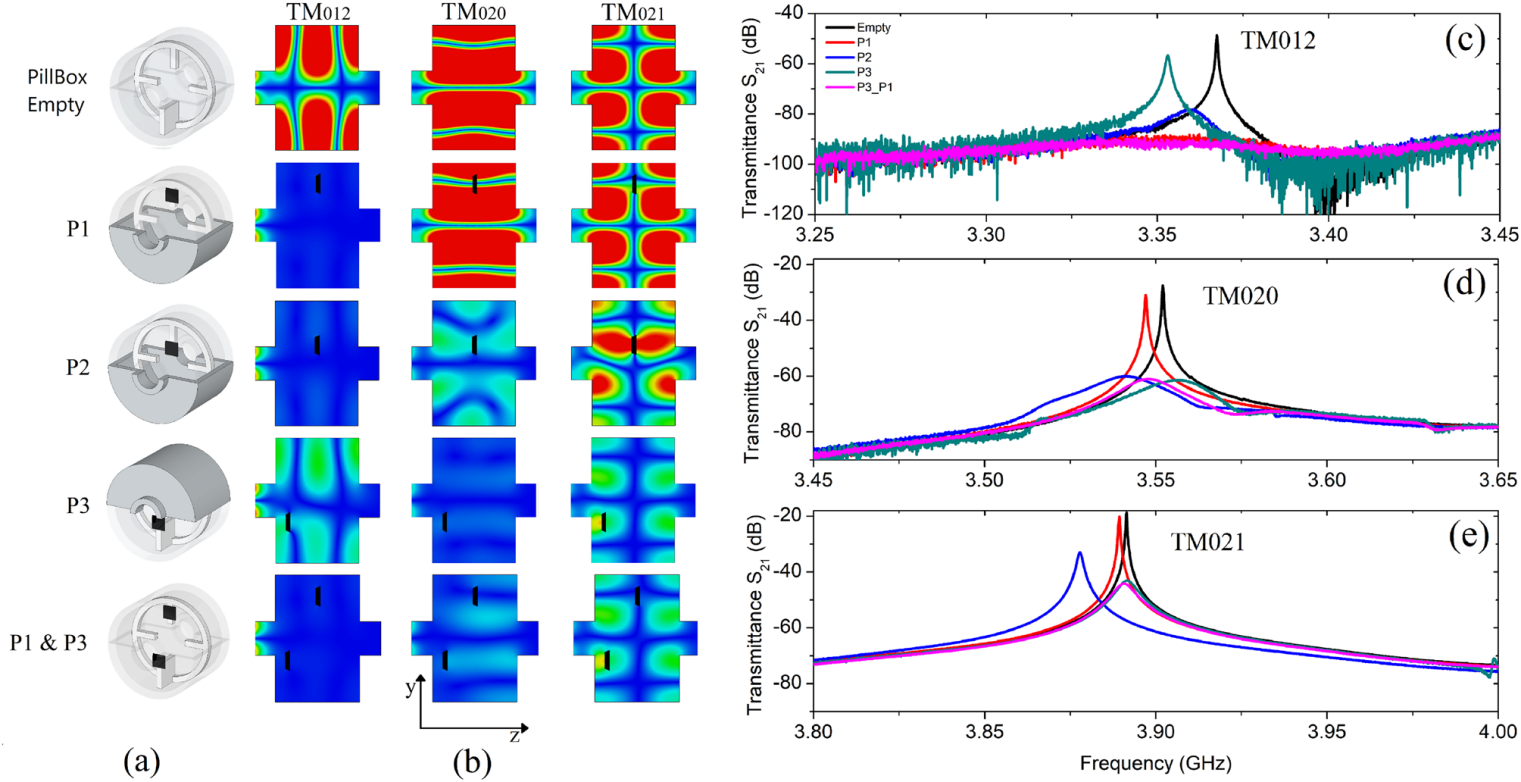
(**a**) Pictorial representation of the simulated overall structure with the different pyramid positions, namely: empty cavity, cavity with square frustum in P1, in P2, in P3, in P1 and P3. (**b**) Section view of the simulated magnetic field distribution inside the cavity (false color scale). Each column represents for the resonant modes under investigation (${\text {TM}}_{012}$, ${\text {TM}}_{020}$, ${\text {TM}}_{021}$) the field variation as a function of the MM position. Each row shows the three mode field patterns for the different configurations. (**c**) Measured ${\text {S}}_{21}$ transmission parameter for ${{\text {TM}}}_{012}$ changing the absorber position: empty (black), P1 (red), P2 (blue), P3 (green) and P1 and P3 (magenta); (**d**, **e**) the same for ${\text {TM}}_{020}$ and ${\text {TM}}_{021}$ respectively.

Resorting to the simulated model, we analysed the EM field distribution of the pillbox TM modes falling within or close-by the absorption bandwidth. The magnetic field amplitude maps for the three targeted empty cavity modes have been evaluated and shown using a false color scale in Fig. 3b (first row of the field distribution plots), from where one can identify at least one region for each mode where the **H** field intensity is at its maximum. Following this analysis, experiments were conducted in order to measure the cavity transmittance inserting only one absorber in three different positions, P1 and P2 along the cavity radial central axis, P3 as P2 but longitudinally shifted. The pyramid positions are displayed in the pictorial representation of Fig. 3a.

For the above defined metamaterial positions we evaluated the magnetic field distribution for each resonant mode and compared it with the corresponding empty cavity result (see Fig. 3b from second to fifth row of the field distribution plots). The first position P1 corresponds to a local maximum of the magnetic field for the ${\text {TM}}_{012}$ mode. The second investigated position P2 is closer to the cavity centre and well inside the region of high magnetic field intensity for the mode ${\text {TM}}_{020}$, representing in this way a good compromise for the damping of both ${\text {TM}}_{012}$ and ${\text {TM}}_{020}$ modes. The third position P3 falls in the high intensity region of both ${\text {TM}}_{020}$ and ${\text {TM}}_{021}$ modes.

Figure 3c–e display the measured transmittance ${\text {S}}_{21}$ of the empty pillbox cavity (black curve) compared with the same structure loaded with the pyramidal absorber in the three selected positions (P1, red curve, P2, blue curve, and P3, green curve) for the three investigated modes respectively. Results are plotted in three separate graphs, for the sake of clarity only. When the truncated pyramid is placed in the P1 position it selectively damps the ${\text {TM}}_{012}$ mode: the resonance at 3.37 GHz is completely absorbed (signal drops from $-48$ dB with more than 42 dB attenuation) whereas the other two resonances at higher frequencies present only a very small red shift. This is also clearly seen from the simulated field maps (Fig. 3b, P1): the ${\text {TM}}_{012}$ mode completely disappears, whereas the distribution of the modes ${\text {TM}}_{020}$ and ${\text {TM}}_{021}$ remains unchanged. In the second configuration (P2) both the ${\text {TM}}_{012}$ and ${{\text {TM}}}_{020}$ modes are absorbed, with signal intensity decreasing from $-48$ to $-78$ dB and from $-27$ to $-60$ dB respectively. The ${\text {TM}}_{021}$ is also somehow affected, but with 14 dB absorption only (signal decreasing from $-19$ to $-33$ dB). As before, the analysis of the field map (Fig. 3b, P2) confirms that for the targeted modes the intensity is proportional to the absorption observed in the ${\text {S}}_{21}$ plot, with strong field disruption of the first and second modes and only a moderate effect on the third mode. Moving the absorber in P3 position, it damps, albeit at a moderate extent, both ${\text {TM}}_{020}$ and ${\text {TM}}_{021}$, the mode ${\text {TM}}_{012}$ instead keeps its field distribution even though with a reduced intensity (Fig. 3b, P3). Under this configuration, an absorption of 8, 34, and 24 dB is measured for ${\text {TM}}_{012}$, ${\text {TM}}_{020}$ and ${\text {TM}}_{021}$ respectively, in agreement with simulation plots.

By analysing the results, it is apparent that the microwave absorption of the first two modes, ${\text {TM}}_{012}$ and ${\text {TM}}_{020}$, can be slightly modulated moving the pyramidal metamaterial along the radial axis from P1 to P2 (see Fig. 3c,d, respectively). The third involved mode ${\text {TM}}_{021}$ instead is strongly affected only when the structure is placed in P3, since positions P1 and P2 are both far from the local maximum field (see Fig. 3e). Given the correlation between MM position and mode damping, in order to maximize the overall absorption the pillbox was loaded with one square frustum placed in P1 (mainly targeting ${\text {TM}}_{012}$), and a second one placed in P3 (targeting ${\text {TM}}_{020}$ and ${\text {TM}}_{021}$). Under this configuration, the experimental ${\text {S}}_{21}$ graph shows that all TM modes falling within the absorption band of the MM present a remarkable attenuation (magenta curves in Fig. 3c–e). We measured a reduction of 42, 34, and 25 dB for the ${\text {TM}}_{012}$, ${\text {TM}}_{020}$ and ${\text {TM}}_{021}$ amplitude resonances respectively, with a corresponding effect on the mode distribution going from a relatively strong to a moderate magnetic field attenuation (Fig. 3b, P1 and P3).

To better quantify the metamaterial damping efficiency we extracted the resonance frequency ${f}_{\text {res}}$ and the loaded quality factor ${\text {Q}}_{\text {load}}=f_{\text {res}}/\Delta f(3dB)$ from the measured transmission spectra for each resonant mode and for each pyramid position. The unloaded quality factor ${\text {Q}}_{0}$, given the symmetry of the coupling on the two ports, is evaluated resorting on the formula:

1 $$\begin{aligned} {\text {Q}}_0 = \frac{{\text {Q}}_{\text {load}}}{1-{\text {S}}_{21}(f_{\text {res}})} \end{aligned}$$

Table 1 summarizes the retrieved ${\text {Q}}_{0}$ values of the targeted modes for the different configurations under investigation, compared to the empty cavity.

**Table 1:** Experimental ${\text {Q}}_{0}$ values of the targeted modes in the empty pillbox cavity and including the pyramidal absorbers in the different configurations under investigation.

	PillBox Empty	P1	P2	P3	P1 & P3
${\text {TM}}_{012}$	9400	N/D	450	3100	N/D
${\text {TM}}_{020}$	11,300	7800	210	220	270
${\text {TM}}_{021}$	12,800	10,800	2500	710	650

## Discussion

The combination of microwave transmission measurements and full-wave numerical simulations proves that pyramidal absorbers having an overall “electrical volume” (volume normalised to the cubic wavelength) lower than 1% can effectively damp different cavity modes. The absorbing effect strongly depends on the square frustum position inside the resonant structure, since the coupling with the pillbox affects the efficiency of the metamaterial as damping element. Closed cavities usually present high Q resonances, therefore in order to have a total damping for a given mode it is crucial to locate the absorber in the region where the magnetic field intensity is at its highest. This is especially observed for the ${\text {TM}}_{012}$ mode. Indeed, starting from a ${\text {Q}}_{0}$ value of approximately $10^4$ in the empty cavity (see Table 1), when the metamaterial is placed in position P1 the quality factor is no more measurable since no resonance is observed in the transmission measurements. The field distributions of ${\text {TM}}_{020}$ and ${\text {TM}}_{021}$ modes instead are only marginally affected by the presence of the absorber, with a maximum reduction of 30% in the quality factor value, likely to be ascribed only to the overall losses in the truncated pyramid. Moving the structure in P2 (high field for ${\text {TM}}_{020}$, moderate field for ${\text {TM}}_{012}$), a clear perturbation in the field distribution and a very strong absorption is observed for both modes. The corresponding ${\text {Q}}_{0}$ factors are lowered by 98% and 95% with respect to the empty cavity values. In this configuration however the ${\text {TM}}_{021}$ mode is affected too, with a ${\text {Q}}_{0}$ value decreasing by 80%. Placing the square frustum in P3, results show a strong damping (95% or larger) for ${\text {TM}}_{020}$ and ${\text {TM}}_{021}$ and a relatively moderate attenuation (65%) for ${\text {TM}}_{012}$. By combining two truncated pyramids (P1 and P3 configuration), the damping effect can be efficiently extended to all three modes. In this case we observe—as expected—a total damping of the ${\text {TM}}_{012}$ mode and a decrease by 98% and 95% in the ${\text {Q}}_{0}$ value for the ${\text {TM}}_{020}$ and ${\text {TM}}_{021}$, respectively (see Table 1).

This behavior clearly shows how the absorption features of the MM depend on its relative position inside the cavity. The mode analysis reported in Fig. 3 underlines how the EM response of the pyramidal metamaterial, and consequently its absorption capacity, is correlated to the intensity of the magnetic excitation field, depending on the cavity modes in our case. The working principle of this type of absorbers relies on the EM wave propagating on the metal-dielectric interface, which is proportional to the incident wave intensity. The induced magnetic field is caused by the antiparallel surface currents at two neighboring layers and is located in the center, since here surface currents are stronger than those at side^34^.

**Figure 4:**
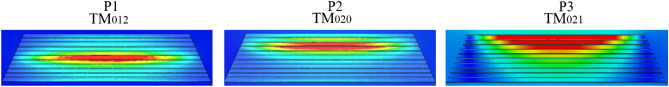
Magnetic field distribution (false color scale) of the ${\text {TM}}_{012}$, ${\text {TM}}_{020}$ and ${\text {TM}}_{021}$ modes inside the truncated pyramid, when it is placed in positions P1, P2 and P3 respectively. The field patterns are obtained on the central cross section in the y–z plane.

The spatially selective and broadband response operated by the MM can be better understood looking at the magnetic field distribution inside the multilayered structure when placed in the pillbox. The total absorbing frequency range is due to the excitation of adjacent layers, with the lower and upper edge of bandwidth defined by the intrinsic resonant frequency of the pyramid bottom and top layers respectively. Going from lower to higher frequencies and for each cavity mode, Fig. 4 shows how the magnetic field (false color scale) is clearly trapped in different resonant layers of the absorbing structure, proving that the absorption band of multilayered structure is due only to the magnetic resonance. It is evident therefore that the most effective damping is reached in those positions where magnetic field is at its maximum for the modes under scrutiny. In the case of ${\text {TM}}_{012}$ and ${\text {TM}}_{020}$ modes, the absorption in the MM is primarily from the median to the upper section. Moving to higher frequencies, for the ${\text {TM}}_{021}$ case, layers near the pyramid top surface are activated since this mode, according to simulations, lies close to the band upper edge. This implies that for each square frustum position, as a consequence of the different cavity mode field distributions, the resonant EM excitation differs from one layer to the other^17^.

Field maps actually confirm that a strong damping of the cavity modes happens only when their resonance frequencies are well within the intrinsic absorption bandwidth of the metamaterial. Nevertheless, measurements put in evidence that mode mitigation for the ${\text {TM}}_{021}$ resonance is realised when the metamaterial is placed in position P3. A possible explanation lies in the large ${Q}_{0}$ value (well exceeding $10^4$) and the corresponding field intensity for this mode in the pillbox cavity, which is high enough to induce surface currents in the upper layers and consequently the excitation of an efficient absorption resonant mechanism. Besides that, some broadening of the experimental frequency spectrum in principle cannot be excluded, likely coming from the unavoidable errors in sample fabrication.

It should be stressed that the absorptive properties of the metamaterial selectively depend not only on its relative position inside the cavity but also on the relative direction of the incident EM wave. In fact, different TM modes have different magnetic field distribution which changes along the cavity, with a component orthogonal to the multilayered structure that in general may not be neglected. However, the square frustum design is very robust with regard to wave impinging direction, ensuring that both absorption level and bandwidth are almost unaltered even when the incidence angle reaches $60^{\circ }$^16^.

In conclusion, we realised a selective absorber for cavity mode suppression in the range 3–4 GHz by inserting a properly designed metamaterial in different positions of a pillbox resonant structure.

Mitigation or suppression of undesired (parasitic or higher order) modes is mandatory in many resonant systems, especially in accelerating structures where high quality particle beams are required. Our study paves the way to a completely different approach in dealing with mode damping in such structures. Instead of widening as much as possible the MM bandwidth by way of design, one can resort to single pyramidal metamaterials, tailored to one or more adjacent specific modes, effectively performing as a broadband absorber with both high efficiency and small volume.

## Methods

The metamaterial was fabricated employing 11 layers of 200 $\upmu \hbox {m}$ annealed copper grown on top of a FR-4 substrate 400 $\upmu \hbox {m}$ thick. The structure was first assembled hot gluing the layers and then mechanically shaped as a truncated pyramid.

As test cavity, we employed an aluminium pillbox whose dimensions are: length $100~\hbox {mm}$, radius $75~\hbox {mm}$, with incoming and outcoming pipes having length $25~\hbox {mm}$ and radius $20~\hbox {mm}$. Coupling ports are realized using a copper wire (length $\sim 3~\hbox {mm}$, radius $0.5~\hbox {mm}$) soldered to an N-type connector. The metamaterial holder is an annulus with four symmetric arms, made of Rohacell®HF31.

The absorbing structure was studied using frequency domain CST Microwave Studio™, assuming dielectric constant 4.3, loss tangent 0.025 for FR-4, and electric conductivity $5.8\times 10^7$ S/m for copper. Stand-alone numerical simulations were performed by applying perfect magnetic and electric boundary conditions on the x- and y-direction respectively, while open boundary conditions were imposed on the z-direction, where the waveguide ports are placed. As for the cavity-pyramid structure simulation, we used an Al cavity (conductivity $3.56 \times 10^7$ S/m) with same dimensions of the test cavity and a Rohacell®(dielectric constant 1.04, loss tangent $< 0.0002$ @2.5 GHz) holder. In order to retrieve the field distribution inside the pillbox, electric and magnetic field monitors have been set in correspondence of each cavity resonant frequency without and with the absorbing structure inserted in different positions.

Calibrated measurements were performed using a 2-port Vector Network Analyser (VNA) Rohde & Schwarz ZNB 20 in the frequency range between 1 and 4 GHz.
